# Professional ethics: the case of neonatology

**DOI:** 10.1007/s11019-018-9863-9

**Published:** 2018-09-07

**Authors:** Michal Stanak

**Affiliations:** 1 Ludwig Boltzmann Institute for Health Technology Assessment, Vienna, Austria; 2 Department of Philosophy, University of Vienna, Vienna, Austria

**Keywords:** Neonatology, NICU, Virtues, Ethics support, Organization, Ethics committees

## Abstract

Neonatal professionals encounter many ethical challenges especially when it comes to interventions at the limit of viability (weeks 22–25 of gestation). At times, these challenges make the moral dilemmas in neonatology tragic and they require a particular set of intellectual and moral virtues. Intellectual virtues of episteme and phronesis, together with moral virtues of courage, compassion, keeping fidelity to trust, and integrity were highlighted as key virtues of the neonatal professional. Recognition of the role of ethics requires a recognition that answering the obvious question (what shall we do?) does not always suffice. Acknowledging the tragic question (is any of the alternatives open to us free from serious moral wrongdoing) and recognizing the ethical dilemmas, where the lines between right and wrong are blurred, leads to actions taken towards establishing ethics frameworks to support decision-making. In neonatology units, such organizational support can help in allowing the team members to recognize the ethical dilemmas, avoid moral distress, and improve team cohesion and the quality of care provided. Only when the organizational structure allows ethical dilemmas to be recognized, adequate decisions can be made.

## Introduction

In all situations of choice, we face what Nussbaum calls the obvious question: what shall we do? Sometimes, however, we also face, or should face, what she calls the tragic question: is any of the alternatives open to us free from serious moral wrongdoing (Nussbaum 2000)? The mere consideration of costs and benefits—as in a cost-benefit analysis (CBA)—helps answer the obvious question, but it often obscures the presence of the tragic one by suggesting that the obvious question is the only pertinent one. CBA indeed helps us figure out, among the options open to us, which one contains the largest net measure of good. However, CBA does not encourage us to divide the alternatives into two distinct classes, those that involve serious moral wrongdoing and those that do not (Nussbaum 2000). This distinction, however, is important as it makes us engage in a form of ethical reasoning that is distinct from the mere CBA. In the situations of neonatal intensive care unit (NICU) decision-making, it does not always suffice to find the answer to the obvious question only. At times, particularly in the ethically intense situations around the *limit of viability*, the tragic question concerning transgression of the moral law is at stake.

This experience of the tragic moral dilemma to which there is no clear solution poses a particular challenge to professional ethics of NICU teams. Of course, the ethics related aspects of the work in a NICU are by no means confined to the experience with the *limit of viability*, but the presence of ethics is more pronounced in the situations of tragedy that sometimes occur around this limit. The experience of the tragic is, however, not exclusive to the work in the department of neonatology or to clinical medicine at large. I want to suggest, however, that in neonatology, the experience of tragedy requires special attention. The reason is that NICU professionals operate around the *limit of viability* with vulnerable infants and parents as surrogate decision-makers. They make decisions about end of life right at its beginning at the age when technologies can prolong life without guaranteeing its quality. Hence, the main reasons that bring about tragedy are clinical uncertainty and ambiguity about best interest.

I believe that virtue ethics has a role to play in addressing the experience of tragedy and so in the following discussion, I explore the applicability of the virtue ethics framework to professional ethics using the case of neonatology. Having a virtues character has been argued to be a requisite for ethical medical practice in general (Pellegrino 2009). Pellegrino puts forth a particular set of virtues for the clinical profession as such, which are compatible also with the case of neonatology. However, I want to argue that for a NICU professional to develop as a moral agent, the organizational culture providing ethics support when the experience of tragedy is imminent is a prerequisite. In terms of the structure of this essay, I am going to explain the challenges that make the moral dilemmas at NICUs tragic, elaborate on the set of virtues required from the NICU professional, and conclude with stressing the need for the organizational culture to recognize the presence of tragedy.

## Ethical challenges at NICUs

### Background

The *limit of viability*, the issue at heart of this essay, is defined as the point in foetal development at which the infant has a reasonable chance of extra-uterine survival (Ehrenkranz and Mercurio 2017). This definition of the limit changes over time due to improvements in treatment and the resulting improvements in outcomes that differ between countries (Zeitlin et al. 2013). Currently in high-income countries, there is a considerable consensus that with an active intervention, most infants born after 25 weeks and 0 days (25 + 0) of gestational age (GA) will survive, while there is little chance for survival and survival without severe impairment in infants born below 22 + 0 weeks of GA (Ehrenkranz 2017). Around the limit, particularly in the *grey zone* of weeks 23 + 0 and 24 + 6, shared decision-making with parents is the strategy of choice. The probability of survival and survival without impairment increases significantly over these few weeks, thus considered the *limit of viability*.

Determining this point with as much precision as possible is important in order to prevent inflicting unnecessary burden on the infant and the family on the one hand (*non-maleficence*), yet to give sufficient chances for survival to the infant on the other hand (*beneficence*). Stratification of decision-making is outlined in Table 1. Zones B and C, the *grey zone*, represent the category of decisions in which balancing the principles of *beneficence* and *non-maleficence* is particularly challenging.

**Table 1:** Stratification of decision-making at the limit of viability (Berger 2011)

Zone	Intensive care	Burden of intensive care	Comment
A	Not indicated	Not acceptable	Parents cannot insist on an unreasaonable intervention
B	Not recommended, but acceptable in individual cases	Likely not to be acceptable	Parental authority should be respected—*zone of parental discretion*
C	Conditionally recommended, but non-initiation acceptable in individual cases	Likely to be acceptable	Parental authority should be respected—*zone of parental discretion*
D	Recommended	Acceptable	Parents cannot reject interventions that are in the infant’s *best interest*

In situations where there exists an alternative open to us that is free from serious moral wrongdoing, which is the clearly the case in zones A and D, the obvious question suffices. That is not true in the *grey zone* (zones B and C). At this point, issues concerning uncertainty and best interest are the main sources of tragic moral dilemmas for NICU professionals.

## Uncertainty

### Assessment of baseline and outcome data

It is the knowledge of baseline and outcome data that provides the frame for discussions about active care versus comfort (palliative) care of NICU infants. The assessment of baseline data, however, is marked with serious uncertainty. Foetal weight can vary as much as 15% and GA estimates vary depending on the assessment tools used (Kett 2015). Maternal dating based upon last menstrual period rarely underestimates the GA, whereas crown to rump measurements have an accuracy of ± 3 days, and the New Ballard Exam was shown to overestimate GA by 2 weeks, with range of ± 4 weeks (Leuthner 2014). Also, the neonatologist’s ability to predict survival based on baseline appearance data and early response is argued to be poor (Brett 2010). And, the assessment of outcome data is subject to gaps in knowledge as, after an educational intervention, NICU professional were more prone to resuscitate regardless of GA (Doucette et al. 2015).

### Empirical and ethical uncertainty

Uncertainties about baseline and outcome data couple with the empirical uncertainly. It is unclear what it is like to live through the experience of comfort care, active treatment that leads to death, or active treatment that leads to neurodevelopmental impairment (NDI) (Leuthner 2014). This is further coupled with ethical uncertainty. The lack of clarity in these three categories of data (baseline, outcome, and empirical) invites value judgments to be made by NICU professionals.

For instance, discerning whether an intervention is futile, beneficial, or in the *grey zone* requires a judgment to be made. Whereas quantitative futility implies that an intervention *does not work*, qualitative futility generally means that an intervention is *not worth it* (Dupont-Thibodeau et al. 2014). At times, NICU professionals seem to conflate these two meanings into one and they communicate to the parents their opinion on qualitative instead of quantitative futility. They make a judgment about what the experience of living with a major NDI must be like without having had the experience of it. Furthermore, they make a judgment on what the threshold quality of life (QoL) worth striving for is, assuming that survival with intolerable deficits may be worse than death. This decision, however, should be taken together with the parents, for it is the parents who primarily give meaning to the prognosis. It seems to be clear that below 22 + 0 weeks of GA, an intervention can be considered qualitatively futile (Dupont-Thibodeau et al. 2014), but, depending on the GA estimate, the *grey zone* examples of weeks 23 + 0 and 24 + 6 of GA remain to be hard to discern. The baseline, outcome, and empirical uncertainties invite ethical uncertainty and respective value judgments to decide what is worth and what is not making the decision-making around the *limit of viability* tragic.

## Best interest

Ambiguity about best interest of the infant is one of the key aspects of moral dilemmas encountered at NICUs. It is not clear what exactly best interest is as well as it is not clear whose best interest is to be decisive. This ambiguity makes the alternatives of the decision such that none of them is clearly free from serious moral wrongdoing. It may create a tension between members of NICU teams as well as between the NICU teams and parents.

### What is best interest?

The best interest principle is grounded in the idea of *beneficence*. The aim is to find out, lacking the opinion of those concerned, what would the given individuals choose themselves. Yet, due to the empirical uncertainly of what it is like to be a patient at a NICU, it is not fully knowable what is beneficial to a vulnerable infant. For instance, a Canadian study points to the lack of understanding of pain that extremely preterm infants go through while at NICUs. Among other themes, the qualitative study states that NICU professionals recurrently mentioned the subtlety and unpredictability of pain indicators, the complex nature of pain assessment, as well as the uncertainty in the management of pain (Gibbins et al. 2015). Hence, the lack of clear clinical facts on the experience in NICUs makes the best interest principle ambiguous.

Not only are the clinical facts about what exactly is beneficial in part unknowable, but the meaning of the word *best* is also inevitably connected to the person who evaluates the case. Different people may interpret the best interest of the extremely preterm infant differently at different times in history. While almost three decades ago, Down syndrome children were left to die because of their *unacceptable* outcomes, today, life-sustaining interventions are no longer considered optional in this segment of the population. The understanding of disability has changed with respect to Down syndrome and so the doctor’s judgment of what the best interest for a Down syndrome infant is has changed as well. This is the case regardless of the fact that if children with Down syndrome were categorized according to the current NICU categorization tools for long-term outcomes, they would be classified as having profound impairments. Their IQ averages below 50, many cannot live independently, and they often die in early adulthood (Dupont-Thibodeau et al. 2014). Thus, putting the NICU challenges into perspective, the future understanding of disability may change and hence influence the today’s judgment of NICU professionals concerning best interest. Therefore, in order to prevent moral wrongdoing, as Berger suggests, the decision to withhold or withdraw life-sustaining therapies needs to be motivated by the desire not to inflict unnecessary suffering on the extremely preterm infant and not by the wish to prevent survival with disabilities (Berger 2011).

### Whose best interest?

When deciding within the *grey zone*, parents as well as NICU professionals are expected to regard the best interest of their newborn child in the first place. But, this distinction between the best interest of the newborn and the best interest of the family is overly individualistic and hence questionable (Leuthner 2014). The best interest principle calls for negating all other interests except for the infant’s self-regarding interest. However, when focusing on the infant’s best interest individually, one tends to put it into contrast with best interests of the family (Leuthner 2014). Those, however, are interrelated and so what is best for the family has the tendency to be also best for the infant. Also for this reason, decisions within the *grey zone* tend to be left within the *zone of parental discretion* as the non-individualistic nature of the best interest principle puts the objective appeal to it into question (Gillam et al. 2017).

The above outlined two challenges make the experience of NICU professionals at the *limit of viability* fall under the category of tragedy because at times, none of the options available is free from serious moral wrongdoing.

## Professional ethics

The professional ethics approach for analysing the normatively sensitive topic of NICU decision-making was chosen for the following reasons. Firstly, the NICU context very clearly highlights the role of agents, NICU professionals, in the value-laden process of decision-making. Particularly, the cases around the *limit of viability* show the need for prompt and tailored decisions that must be made on the spot against the backdrop of the list of above outlined uncertainties and ambiguities. NICU professionals thus must take a value stand when facing these challenges and discern what moral principles to follow and what moral principles to forgo in what situations. This puts their character into focus. Secondly, in the body of literature on ethics of NICUs, almost no mention of the role of virtues was found. Rights-based, utilitarian, or principlistic approaches dominate the field and none of them puts enough focus on agents who make moral decisions on the spot against the backdrop of their specific context. And thirdly, following the conviction of A.MacIntyre that virtues develop in practices (MacIntyre 2007), I want to suggest that it is not only clinical judgment, but also moral judgment that should come with expertise and that is required for acting well in the clinical practice of neonatology. There are character traits, virtues, that are at stake here and those are intellectual virtues of *episteme* and *phronesis* and moral virtues of *courage, compassion, fidelity to trust*, and *integrity*.

### Intellectual virtues

The inevitability of uncertainty that NICU professionals at times experience further complicates the above outlined issues with discerning the best interest. For that reason, possessing the intellectual virtues of *episteme* and *phronesis* is key. Only the professional with quality understanding of the scientific, *epistemic*, data can build on this factual knowledge and work towards the goal of clinical practice in general and neonatology in particular - the good of the patient (MacIntyre 2007). Furthermore, because *phronesis* links intellectual and moral virtues, it is essential for the NICU professional for discerning the right course of action with regards to both scientific data as well as moral principles. It refers “to the reasoned capacity to act with regard to the things good for the patient—both technical and moral” (Pellegrino 2009). As put by the US President’s Council on Bioethics: “There are no simple formulae to guide us, no algorithms for calculating the relative weights of benefits and harms. Seeking the best care possible will always require wise and prudent [*phronetic*] judgment of the people on the spot.” (President’s Council on Bioethics 2005). The *phronetic* judgment is key also in the use of the following set of moral virtues particularly required in the practice of neonatology.

### Moral virtues

Ambiguity about what the best interest is, combined with the unavoidability of surrogate decision-making bring about challenges to the professional ethics of the members of NICU teams. In the *grey zone*, these challenges occur at the backdrop of parental decision-making preferences. As a US qualitative study suggests, parents identified two factors that influence their preference to delegate decisions to the NICU team: high level of urgency and high level of medical expertise required (Weiss et al. 2016). Parents also identified four factors associated with their preference to retain control over decisions: high-perceived risk, high personal experience with the decision, involvement of foreign bodily fluids, and similarity to decisions that they perceived as part of the normal parental role (Weiss et al. 2016). Furthermore, according to an Austrian qualitative study, parents sometimes want the team to *do everything for the baby* (regardless of the suffering inflicted), while other times, parents want to quit active treatment (regardless of the infant’s chances for meaningful survival) (Stanak and Hawlik 2017). Complications arise when there is a conflict between what the NICU team thinks is the best interest for the infant and what parents think. Rarely do parents want to stop the treatment and the team thinks the infant has good chances for meaningful survival (Stanak and Hawlik 2017). Parents tend to want to prolong life, but the professional conflict also arises when it comes to prolonging life that has little chances for meaningful survival (that is now possible thanks to advances in the technology) (Stanak and Hawlik 2017). Thus, as the aim of the NICU team is the best interest of the child, which may get in conflict with parental wishes or which may cause a disagreement within NICU teams, it is in these particularly heated situations that the character of NICU professionals is put at stake.

When such situations of disagreement do occur, it seems that there is no alternative that is free from serious moral wrongdoing. On the one hand, the NICU professionals interfere with the *zone of parental discretion* that lays the entire weight of the decision on the parents (Gillam et al. 2017), while on the other hand, the NICU professionals face the demand of their own *act of profession* that commits them to serve the best interest of the infant (Pellegrino 2009). As infants at NICUs cannot make autonomous decisions for themselves (Doroshow et al. 2000), the legal guardians are expected to be the surrogate decision-makers and decide on their behalf (Berger 2015).

In practice, doctors seem to have the tendency to accept family’s refusal of resuscitation of an extremely preterm infant even if they think that resuscitation is in the infant’s best interest (Janvier et al. 2008). In an Australian survey, 96% of doctors were willing to comply with families’ wishes to withhold intensive care, despite 77% of them believing that resuscitation would be in the infant’s best interest (Mills et al. 2015). This poses a challenge to the NICU profession. Because parents are not always in the right to decide (Leuthner 2001), as they face a decision they presumably never anticipated and so they may be ill prepared to make such a decision about their infant (Weir et al. 2011), the clinicians may be arguably more competent to make the decision amidst the tragic moral dilemma. Knowing when to step in and when to override parental decision, however, presents a professional conflict that requires the virtue of *courage* to challenge the parents and the virtue of *phronesis* to discern when to apply what course of action.

Knowing when to step in to decide is made even more complicated by the fact that the virtue of *compassion* is reported to be the distinguishable character trait of NICU professionals. Compared to the obstetric personnel, neonatologists are in general more prone to be interventional within the *grey zone* in weeks 23 + 0 to 24 + 6 of GA (Chan et al. 2006). It is a reoccurring topic in the literature that neonatologists are different from obstetricians in their approach (Chan et al. 2006). The basic difference is that neonatologists seem more pro-life, pro-caring for infants with complications, and are more hopeful that intensive care will not lead to further complications. Obstetricians are said to be more focused on the best interest of the parents, in particular the mothers, and on avoiding impairment (Stanak and Hawlik 2017).

A further complication arises from the fact that infants and their families tend to spend months at the NICUs, which makes the relationship with the NICU professionals more individual and personal. It is particularly the case with the NICU nurses who spend a considerable amount of time with parents at the infant’s bedside and thus often act as family advocates (Green et al. 2015). An Australian study found that NICU nurses often find themselves in challenging situations where they have to *keep secrets* from the parents. Due to the challenge created by the trust given to them by the parents and the nurses’ up-to-date knowledge about the health state of the infant, the nurses reported the experience of fear of inadvertent disclosure, fear of parents being unable to cope with potentially catastrophic news, or fear of knowing of a burden that could damage the trust between them and the parents (Green et al. 2015). These situations pose a challenge for the character of NICU nurses with respect to *keeping fidelity to trust* given to them by parents and their own *integrity*.

### Organizational culture

Discerning the right course of action in the NICU is predicated by a certain degree of freedom to act in accordance with one’s decision. Understanding virtues as character traits that manifest themselves in professions and that dispose the NICU professionals to habitually act well puts an emphasis on the organizational structures. I want to suggest that the organizational structures play a vital role in allowing the NICU professionals to develop in their professional virtues. For that reason, the organizational structures ought to recognize the role of tragedy in the NICU decision-making and hence recognize the ethics-related aspects of the work in NICUs.

Recognizing the presence of the tragic question can have impact on both the team as well as the individual. In terms of the individual NICU professional, recognizing the tragic question may help with the experience of moral dilemmas and, at times, moral distress and thus allow the members of the NICU team to acknowledge and work with the dilemmas individually. In case of NICU teams, recognizing the fact that there is a separate tragic question that is different from the obvious question leads to the recognition of the role of ethics support in the NICU decision-making environment.

#### NICU individuals

A reoccurring theme in the literature is that the bedside experience of the NICU nurses leads to the experience of futility (Stanak and Hawlik 2017). Nurses are close to the suffering of vulnerable infants and thus see the futility of intensive care. It is partly due to the fact that while 50 years ago, the majority of neonatal deaths occurred regardless of the best efforts of NICU teams, today, the majority of neonates die after the life-sustaining interventions are withdrawn, not withheld (Mills et al. 2015). As reported in a UK qualitative study among NICU nurses, the use of advanced technology brought with it an increased number of moral dilemmas (Gallagher et al. 2011).

Furthermore, being so close to suffering and thus going through the normative tension inherent to the situation may make the nurses experience moral distress (Catlin 2008). One of the most common causes of distress in other areas of medicine lies in supporting patients at the end of their lives when comfort care would be more humane. The same is the case for NICU nurses as well. Nurses report that they struggle with causing suffering through carrying out active treatment when they could comfort instead and so they are more prone to withhold resuscitation altogether (Catlin 2008). The experience of moral distress, however, differs from the experience of a moral dilemma in that the nurses think they know what the right course of action is, but the institution and other co-workers (doctors in the case of NICUs) create obstacles for them to act according to their conscience (Jameton 1993). The fact that NICU doctors tend to be more hopeful and thus interventional while the NICU nurses tend to want to withhold active interventions creates a tension point. This tendency is confirmed by a Swiss survey among NICUs that indicates that 35% of doctors and 64% of nurses stated that some infants were treated too intensively (Klein 2016). Such tension points to the presence of tragedy and hence to the need to explicitly recognize the role of ethics in NICU decision-making.

#### NICU teams

Discussing the virtues of NICU professionals is secondary to allowing NICU professionals to be open about the role of ethics in their profession. I want to suggest that insofar as the role of moral enquiry is recognized by the respective organization, the normative tension can be eased via the use of mechanisms of ethics support. The same Swiss survey mentioned above further states that 50% of doctors and 78% of nurses wished for more group discussions about ethics-related issues (Klein 2016). It suggests that the topic of ethics receives less attention than it is supposed to. However, to speak openly about ethics in the NICU teams, the organizational set up must be such that it is allowed. The challenges with such openness to ethics can be manifold. Starting from the background legal context that sets the broad boundaries of the ethics debate, to the general societal recognition of the role of ethics, and individual attitudes of clinical professionals to the field of ethics. An Austrian qualitative study further states that bringing the conflicts from the personal to the value level poses a challenge, as well as the individualized and competitive nature of the medical profession that, compared to other professions, sometime lacks the recognition of the need for building of a team culture (Stanak and Hawlik 2017).

Support from ethics committees, in-house supervision, and ethics moderation of team discussions are the current good practice mechanisms that help bring the ethics related aspects of the work in NICUs to be explicit. While the practice of ethics committees that support NICU decision-making (formally or informally) is fairly common (Nuffield Council on Bioethics 2006; Stanak and Hawlik 2017), in-house supervision structures depend on the particular hospital set-up. In the Austrian context, ethics committees at times serve as a moderation mechanism that helps resolve the NICU team tensions via moderating team discussions. In this way, members of ethics committees are send to the neonatal departments with the aim of bringing the issue from a personal conflict to a conflict at the value level for the sake of improving the decision (Stanak and Hawlik 2017). Such moderation helps minimize the variety of opinions in the team and thus allows the team to go with one opinion to support parents in the process of ethics consultation. An Italian qualitative study further states that there is a need for formal ethics consultation and ethics training programmes for neonatal professionals for the sake of improving the quality of care delivered (De Panfilis et al. 2018).

The correlation between ethics consultation and quality of care provided is also the result of a US survey among neonatologists (Weiss et al. 2007). A similar point is also supported by a Canadian survey that suggests that organizational culture has an impact on the improvement of quality of care at a NICU (Mahl 2015). Since ethics support may improve the organizational culture through making the ethical tension points become transparent and to some extent solved within the teams, it may indirectly improve the service delivered. The survey concludes that group oriented culture leads to better outcomes, however particularly in Canadian NICUs, hierarchical culture is associated with even better patient outcomes (Mahl 2015). For that reason, ethics consultations from the outside, but also the need of team buildings for NICU professionals within teams, are necessary to support the team cohesion for the sake of improved decisions.

If the NICU management openly recognizes the tragic moral dilemmas that are at times experienced by NICU professionals, it makes it easier for individuals to progress not only as professionals, but also as moral agents. It may have an impact on the team cohesion and, hence, also on the quality of care delivered. Allowing clinical ethics to play a role especially in normatively challenging situations such as those encountered within the *grey zone* at NICUs may lead to improved quality of the team as well as quality of the NICU professional within it.

## Conclusion

Distinguishing between the obvious and the tragic questions is important because it allows us to engage in a form of ethical reasoning that would otherwise be obscured by the presence of the mere CBA. In NICUs, I focused on the experience of tragedy, that is when there is no option that would be free from serious moral wrongdoing, which can occur when deciding around the *limit of viability*. Due to this experience of tragedy that is caused by the high level of uncertainty and ambiguity of the best interest principle, I argued that the case of neonatology requires special attention. In line with the virtue ethics literature, I identified the intellectual virtues of *episteme* and *phronesis* and moral virtues of *courage, compassion, fidelity to trust*, and *integrity* as key. However, I argued that the discussion about virtues can only take place once the organizational structures acknowledge the distinction between the obvious and the tragic and so allow the NICU professionals to engage in the moral enquiry. With this freedom and, ideally, with some form of ethics support, NICU professionals as well as NICU teams can grow as moral agents. Virtue ethics thus has a role to play in addressing tragedy through focusing on the individual agent, the NICU professional, who needs to decide on the spot on how to solve the complicated dilemmas encountered in the NICU profession and who needs ethics support in the process. Only when the organizational structure allows ethical dilemmas to be recognized, adequate decisions can be made.
